# Teaching young GPs to cope with psychosocial consultations without prescribing: a durable impact of an e-module on determinants of benzodiazepines prescribing

**DOI:** 10.1186/s12909-017-1100-3

**Published:** 2017-12-19

**Authors:** Hanne Creupelandt, Sibyl Anthierens, Hilde Habraken, Tom Declercq, Coral Sirdifield, Aloysius Niroshan Siriwardena, Thierry Christiaens

**Affiliations:** 1 Department of General Practice, Primary Health Care Ghent University, Ghent, Belgium; 2 Department of Primary Health Care and Interdisciplinary Care, University of Antwerp, Antwerp, Belgium; 3 Farmaka, Ghent, Belgium; 4 Community and Health Research Unit, School of Health and Social Care, University of Lincoln, Lincoln, UK; 5 Clinical Pharmacology Research Unit, Ghent University, Heymans Institute of Pharmacology, Ghent, Belgium

**Keywords:** Benzodiazepine, General practitioner, Coping with psychosocial consultations, Psychological determinants of prescribing practice, Changing prescribing behavior, E-intervention

## Abstract

**Background:**

Despite guidelines and campaigns to change prescribing behavior, General Practitioners (GPs) continue to overprescribe benzodiazepines (BZDs). New approaches to improve prescribing are needed. Using behavior change techniques and tailoring interventions to user characteristics are vital to promote behavior change. This study evaluated the impact of an e-module on factors known to determine BZD prescribing practice.

**Methods:**

A tailored e-module that focuses on avoiding initial BZD prescriptions (and using psychological interventions as an alternative) was developed and offered to GPs in vocational training. Three self-report assessments took place: at baseline, immediately after the module (short term) and at least six months after completion (long term). Assessed determinants include GPs’ attitudes concerning treatment options, perceptions of the patient and self-efficacy beliefs. Readiness to adhere to prescribing guidelines was evaluated through assessing motivation, self-efficacy and implementability of non-pharmacological interventions.

Changes in determinants were analyzed using the Wilcoxon signed-rank test. Changes in readiness to adhere to guidelines was analyzed using the nonparametric McNemar Bowker test.

**Results:**

A desirable, significant and durable impact on determinants of BZD prescribing was observed. GPs (*n* = 121) underwent desirable changes in their attitudes, perceptions and self-efficacy beliefs and these changes remained significant months after the intervention. Barriers to using a non-pharmacological approach often cited in literature remained absent and were not highlighted by the intervention. Furthermore a significant impact on GPs’ readiness to adhere to guidelines was observed. Participants reported change in their ability to cope with psychosocial consultations and to have tried using non-pharmacological interventions.

**Conclusions:**

Tailoring an e-intervention to target group (GPs) characteristics appears to be successful in promoting behavioral change in GPs undertaking vocational training. Significant and lasting changes were observed in determinants of prescribing BZDs. The e-intervention resulted in a positive impact on participants’ readiness to adhere to BZD prescribing guidance and their coping with psychosocial consultations.

Investigating which mechanisms of change are responsible for the observed effectiveness could help to refine and improve future interventions.

**Electronic supplementary material:**

The online version of this article (10.1186/s12909-017-1100-3) contains supplementary material, which is available to authorized users.

## Background

Benzodiazepines (BZDs) are one of the most commonly used psychotropic drugs to treat conditions such as insomnia and anxiety in primary care. However, long-term use is associated with considerable adverse effects including memory disruption, increased risk of accidents and falls, and dependence [1–4]. Despite guidance advocating use of non-pharmacological, psychological treatments first-line, and using BZD only short-term and if needed, numerous studies have shown that BZDs are overprescribed and chronic use is common [5–9].

Since General Practitioners (GPs) regard prescribing of BZDs as one of the most complex, demanding and uncomfortable tasks in their clinical work [10], it is confusing that GPs continue to prescribe these drugs frequently. Reviews identify a variety of reasons for inconsistent BZD prescribing in primary care [11]. The main concern for GPs is to help the patient: GPs try to manage the tension between minimizing prescribing and their responsibility to help ‘deserving’ patients [10, 12]. In this context it is important to stress that patients also have an impact on GPs’ prescribing decisions [11–15].

Sirdifield et al. [11] developed an explanatory model of processes underlying current prescribing practices of BZDs in primary care. This model can be used to support and evaluate interventions to improve adherence to BZD prescribing guidance. The model emphasizes that prescribing BZDs is a behavioral outcome, determined by several factors. Interventions should not merely focus on acquiring knowledge about BZDs or non-pharmacological alternatives, but should also address other determinants contributing to inconsistent prescribing practices. Thus, training programs should also focus on ambivalent attitudes and perceptions, as they are important determinants of inconsistent prescribing strategies [16].

Most training programs for GPs have focused on helping patients discontinue long-term BZD use [17, 18]. Studies evaluating these interventions generally operationalize improvement in prescribing as lower overall rates of BZD prescriptions.

Although the best way to avoid BZD dependence is by not initiating these drugs [10], fewer interventions focus on initial prescriptions. Evaluative studies are confronted with the complex task of defining prescribing appropriateness and what is meant by an improvement in prescribing practice [18]. Using quantities of BZD prescriptions as an outcome measure, is consequently problematic. Thinking beyond quantification of prescribing and selecting psychological determinants of prescribing as outcome measures makes more sense within this context. Such studies are scarce as an explanatory model of processes underlying prescribing BZD has only recently been developed.

Along similar lines, educational interventions typically focus on acquiring knowledge about correct prescribing [18] and are seldom attuned to the psychological dynamics occurring within GPs. Using behavior change techniques is vital however, when promoting behavior change [19]. There is consequently a compelling case for GP – attuned interventions which focus on avoiding initial BZD prescriptions and using psychological treatments as an alternative.

This study evaluates the impact of an e-module on the readiness of GPs in vocational training to adhere to BZD prescribing guidance. The effectiveness of the e-module is evaluated by its impact on psychological determinants (attitudes, perceptions and self-efficacy beliefs). Furthermore, participants’ readiness to change prescribing practice (motivation, self-efficacy and implementability of non-pharmacological interventions) is assessed.

## Methods

### Participants and process

During a period of 2.5 years (January 2012 – July 2014), all Flemish GPs in vocational training had an opportunity to participate in a free e-module about BZD prescribing. GPs in vocational training are graduate medical doctors that work full-time for two years as a GP trainee under the supervision of an experienced GP. The e-module was publicized on the website of the inter-university center for Flemish GPs in vocational training. This center informs young GPs monthly by email about new educational programs. GPs in vocational training could volunteer to participate by registering for the web-based module. Participants who gave informed consent for participating in the study were asked to complete a self-report assessment when starting and when ending the e-learning module. Six months after completion, participants received an email invitation for completing a post-intervention questionnaire. Non-responders were sent several reminders.

### Intervention

The intervention focuses on avoiding initial BZD prescriptions and using psychological interventions as an alternative (see Additional file 1, [20–24]). The intervention was a web-based program, following the evolution of e-learning as a popular alternative to face-to-face learning, including in continuing medical education. The e-module drew on the existing theory of Self-Determination (SDT [25]), which provides both theoretical grounds and practical guidelines to create motivating learning environments [26, 27]. According to SDT [25], the e-module aimed to fulfil basic psychological needs for autonomy, competence, and relatedness of participants. Furthermore, the intervention attunes to psychological dynamics that are known to determine GPs’ BZD prescribing practices (see below).

### Outcome measures

The intervention was evaluated considering its impact on psychological determinants and readiness to adhere to prescribing guidelines and therefore focusing beyond simply quantifying prescriptions. Assessed psychological determinants comprised GPs’ attitudes concerning treatment options and GPs’ perceptions of the patient, as described in the explanatory model of Sirdifield et al. [11]. This model emphasizes how prescribing BZDs is a behavioral outcome determined by several factors (GPs’ attitudes towards interventions, GPs’ perception of the patient, GPs’ sense of responsibility). Since all trainees participated on a voluntary basis, the third determinant of the explanatory model (sense of responsibility for BZD prescribing practice) was not assessed. Based on the findings that GPs appear to use BZDs to deal with their feelings of helplessness and uncertainty concerning both the doctor-patient relationship and non-drug alternatives for BZDs [10], items assessing GPs’ self-efficacy beliefs (beliefs about capabilities, a psychological determinant of behavioral intentions [28]) were added to the self-report assessments.

Readiness to adhere to prescribing guidelines was evaluated through assessing motivation, self-efficacy and implementability of non-pharmacological interventions. Based on the transtheoretical model (TTM), a frequently used biopsychosocial model to conceptualize the process of intentional behavior change [29, 30], participants were asked to declare whether they intended to make a change, had actually made efforts to change their prescribing practice and state self-efficacy beliefs concerning their ability to change their prescribing practice.

To assess the implementability of alternative, non-pharmacological treatment strategies, participants evaluated non-pharmacological interventions on their meaningfulness and practical usefulness and declared whether they actually used the listed treatment strategies in their practice.

### Assessments

Three self-report assessments took place: one pre-intervention (baseline measurement) and two post-intervention assessments, immediately after completing the module (short-term impact) and months after completion (long-term impact). All three self-report assessments comprised 10 items evaluating psychological determinants of BZD overprescribing in primary care (Table 1). Determinants were assessed using 10 items rated agree/disagree on a 5-point Likert scale. Additionally, items assessing participants’ readiness to adhere to BZD prescribing guidelines (Table 2) were added to the self-report assessments. At each self-report assessment, participants were asked to make a selection of statements that they agreed with, in order to determine where they were situated on the continuum between ‘not prepared to prescribe less’ and ‘already prescribing little sleep medication’.

**Table 1:** Impact on Psychological Determinants of BZD overprescribing (*n* = 121)

		strongly disagree (%)	disagree (%)	neutral opinion (%)	agree (%)	strongly agree (%)	Wilcoxon Z	*p* value
GPs’ attitudes concerning treatment options								
1. The advantages of sleep medication outweigh the disadvantages.								
	Baseline (%)	29.3	53.7	14.6	2.4	0		
	Short term (%)	52.8	36.6	4.1	3.3	3.3	−2.618	.009**
	Long term (%)	55.3	34.2	6.1	1.8	2.6	−3.331	.001**
2. There are no non-drug alternatives for sleep problems that are as effective as drugs.								
	Baseline (%)	18.7	47.2	26.8	5.7	1.6		
	Short term (%)	38.2	47.2	10.6	3.3	0.8	−4.333	<.001*
	Long term (%)	41.2	37.7	14.9	4.4	1.8	−3.346	.001**
3. I don’t have time to treat sleep problems using non-drug therapies								
	Baseline (%)	16.3	51.2	22.8	8.9	0.8		
	Short term (%)	14.6	21.5	25.2	8.9	0	−0.161	.872
	Long term (%)	14	53.5	23.7	7.9	0.9	−0.235	.814
4. The non-medicational treatment of sleep problems is the business of other professionals								
	Baseline (%)	34.1	50.4	10.6	4.9	0		
	Short term (%)	32	48.4	13.9	5.7	0	−1.197	.231
	Long term (%)	31.9	50	10.3	5.2	2.6	−0.997	.319
5. Non-drug treatment of sleep problems needs to be supported with medication.								
	Baseline (%)	26.8	53.7	17.1	0.8	1.6		
	Short term (%)	37.4	52	10.6	0	0	−2.906	.004**
	Long term (%)	45.1	46.9	7.1	0.9	0	−4.145	<.001*
GPs’ perception of the patient								
6. If I do not prescribe medication to a patient with sleep problems, (s)he is dissatisfied.								
	Baseline (%)	0.8	17.9	27.6	50.4	3.3		
	Short term (%)	4.9	35.8	33.3	24.4	1.6	−5.797	<.001*
	Long term (%)	6	34.5	30.2	27.6	1.7	−4.782	<.001*
7. It is difficult for a GP to motivate a patient with sleep problems to choose a non-medicational treatment.								
	Baseline (%)	0.8	17.9	12.2	51.2	17.9		
	Short term (%)	3.3	26	22	42.3	6.5	−4.549	<.001*
	Long term (%)	1.7	22.4	15.5	50	10.3	−1.908	0.056
GPs’ self-efficacy beliefs								
8. When I am not prescribing medication for sleep problems I feel like I am not empathic								
	Baseline (%)	22	51.2	13.8	13	0		
	Short term (%)	33.3	50.4	10.6	5.7	0	−3.154	.002**
	Long term (%)	39.7	41.4	14.7	4.3	0	−3528	<.001*
9. I have the expertise to use non-drug treatment for sleep problems.								
	Baseline (%)	22.8	33.3	23.6	18.7	1.6		
	Short term (%)	0	11.4	33.3	51.2	4.1	−7.217	<.001*
	Long term (%)	0.9	7.9	32.5	54.4	4.4	−7.133	<.001*
10. I often feel overwhelmed when a patient presents with psychosocial problems								
	Baseline (%)	6.5	42.3	21.1	26.8	3.3		
	Short term (%)	10.6	44.7	23.6	20.3	0.8	−2.246	.025*
	Long term (%)	20.7	44.8	22.4	8.6	3.4	−4.611	<.001*

*Within the text, participants who “agree” and “strongly agree” are referred to as “agreeing”. In the same way, participants who reported to “strongly disagree” or “disagree” are generally referred to as “disagreeing”*

*p < 0.05 **p < 0.01

**Table 2:** Readiness to adhere to prescribing guidelines (*n* = 121)

		selected by (%)	Mc Nemar	*p* value
Intention to change				
I do not intend to prescribe less sleep medication.				
	Baseline	0.8		
	Short term	0		1
I intend to prescribe less sleep medication within the next weeks (< one month).				
	Baseline	19.5		
	Short term	37.4	12.971	<.001**
Made efforts to change				
I have tried in the past to prescribe less sleep medication				
	Baseline	23.6		
	Long term	67.2	41.397	<.001**
I have been trying to prescribe less sleep medication for some time (more than 6 months).				
	Baseline	22.8		
	Long term	63.8	38.473	<.001**
Self-efficacy beliefs				
I intend to prescribe less sleep medication* but don’t know how.				
	Baseline	43.1		
	Short term	2.4	42.875	<.001**
	Long term	1.7	43.184	<.001**
I am trying at the moment to prescribe less sleep medication but without success.				
	Baseline	29.3		
	Short term	12.2	13.793	<.001**
	Long term	15.5	7.314.	.007**
I am trying at the moment to prescribe less sleep medication and have succeeded in doing so.				
	Baseline	12.2		
	Short term	47.2	34.588	<.001**
	Long term	62.9	50.766	<.001**

*p < 0.05 ** *p* < 0.01

‘Intention to make a change’ was assessed at baseline and when completing the module (short-time impact). Self-perceived ‘efforts to change’ were assessed at baseline and months after completing the module (long-term impact). Items assessing self-efficacy beliefs concerning one’s ability to change were added at all three assessments.

The two post-intervention assessments comprised items assessing the implementability of six alternative treatment strategies (Table 3). After completing the module, participants evaluated these psychological interventions on their meaningfulness and practical usefulness using a 5-point Likert scale. Six months after ending the module, the non-drug treatment strategies were evaluated on their practical usefulness (again using a 5-point Likert scale) where participants were asked whether they ‘never’, ‘rarely’ or ‘frequently’ used the listed treatment strategies in their practice. In the last questionnaire – administered months after ending the module - participants rated on a 5-point Likert scale whether they felt the E-intervention had changed their practice.

**Table 3:** Implementability of 6 demonstrated alternative treatment strategies (*n* = 121)

		Short term (when ending)		Long term (months later)	
		Meaningful (%)	Useful (%)	Useful (%)	Used (%)
ICE model of communication					
	strongly disagree / never	0	0	0	1.7
	disagree	0	0	0	
	neutral. no opinion / rarely	3.3	11.4	9.3	16.0
	agree	42.3	48.4	56.8	
	strongly agree / frequently	54.5	40.2	33.9	82.4
Sleep hygiene education					
	strongly disagree / never	0	0	0	8.5
	disagree	0	0	0	
	neutral. no opinion / rarely	1.7	4.9	0.9	29.7
	agree	25.6	25.4	35.3	
	strongly agree / frequently	72.7	69.7	63.8	61.9
Stress-vulnerability model					
	strongly disagree / never	0.8	0.8	0.9	29.6
	disagree	2.5	5.7	4.3	
	neutral. no opinion / rarely	10.7	22.1	17.1	39.1
	agree	45.5	31.1	38.5	
	strongly agree / frequently	40.5	40.2	39.3	31.3
Sleep wake diary					
	strongly disagree / never	0.8	0.8	1.7	32.2
	disagree	0	1.6	6.0	
	neutral. no opinion / rarely	4.1	17.2	20.7	49.6
	agree	43.1	42.6	50.0	
	strongly agree / frequently	52	37.7	21.6	18.3
Stimulus control therapy					
	strongly disagree / never	0	0	1.7	37
	disagree	0.8	0.8	1.7	
	neutral. no opinion / rarely	9	13.8	33.9	42.9
	agree	39.3	44.7	33.9	
	strongly agree / frequently	50.8	40.7	28.8	20.2
ABC model					
	strongly disagree / never	0	0.8	1.7	50.9
	disagree	4.9	11.4	16.0	
	neutral. no opinion / rarely	13.8	28.5	29.4	41.4
	agree	55.3	41.5	38.7	
	strongly agree / frequently	26	17.9	14.3	7.8

To consider validity, all items were submitted to an interdisciplinary expert group (GPs, psychologists and a sociologist).

### Analysis

Effects of the intervention were identified by using the assessments within a pretest-posttest study design. Short-term effects were identified using the assessment undertaken when participants completed the module as posttest. The assessment that was sent at least six months after completing the intervention was used as posttest to identify long-term effects.

The impact of the intervention on psychological determinants was analyzed using the Wilcoxon signed-rank test as the nonparametric equivalent to the dependent t-test. This test was used to identify differences on each paired ordinal variable.

Effects on readiness to adhere to guidelines was analyzed using the nonparametric McNemar Bowker test. The McNemar test was used to identify differences on paired proportions on each selected statement as a dichotomous variable.

## Results

### Participants

During a period of two and a half academic years, 33% (266/803) of the Flemish GPs in vocational training used the E-module on a voluntary basis and completed a baseline self-report assessment. Most of them (64%, 171/266, i.e. 21% of 803 potential participants) also completed a self-report assessment when completing the E-module. About 70% (120/171) reported having spent more than 3 h on the E-module and 87% (149/171) chose to spread the intervention in time (as suggested), with a median of 25 days.

One hundred twenty-one GPs in vocational training (45% of the initial 266 participants, i.e. 15% of the 803 potential participants) also completed the follow-up assessment, with a median of seven months between this assessment and the baseline assessment. Only participants who completed all three assessments were included in this study.

76% of the study sample were female and most of the participants were in their late twenties (proportions which are similar to the general population of GPs in vocational training in Flanders). Participants were working in different regions and graduated from different Flemish universities. Assessed base-line characteristics are reported in the tables.

### Impact on psychological determinants

The assessments showed a desirable, significant and durable impact of the intervention for several determinants of BZD prescribing (Table 1). First, the assessments showed enduring changes in GPs’ attitudes concerning treatment options: not only short-term (on completing the module), but also in the longer term (at least six months later), attitudes changed significantly. Participants disagreed more strongly with the statement that the advantages of BZDs outweigh their disadvantages and they judged more strongly that non-drug alternatives are as effective as BZDs. Participants disagreed more strongly that non-drug treatments need to be supported with medication. Neither the idea of lacking time to use non-drug interventions nor perceiving them as the business of other professionals appeared to be a barrier for participants at the baseline assessment. Since no significant impact on these items was observed on the short term nor on the longer term, the E-module did not highlight such barriers.

In addition, the intervention significantly influenced the way GPs perceived sleepiness patients. When completing the module, but also months later, participants were less convinced that it took a medical prescription to satisfy a patient. Although participants agreed less strongly that patients were difficult to motivate in choosing a non-drug treatment when completing the intervention, this effect did not show statistical significance in the longer term (Z = −1.908, *p* = 0.056).

Further, significant and durable changes were observed concerning the self-efficacy beliefs of GPs in vocational training. Not only in the short term, but also in the longer term (at least six months later), participants reported a significantly stronger sense of self-efficacy. Participants disagreed more strongly that they had to show empathy by prescribing and felt more strongly that they had the expertise to use non-pharmacological interventions. When starting the module, 20.3% felt that they had such expertise, while 58.8% reported this months after ending the E-module. Also, participants reported feeling less overwhelmed when a patient presented with psychosocial problems during a consultation. At baseline 30.1% reported feeling overwhelmed, while only 12% reported this in longer term.

### Impact on readiness to adhere to prescribing guidelines

The module showed significant and desirable effects on participants’ readiness to change prescribing behavior (Tables 2 & 3). Firstly, the intervention appeared to have a significant and positive effect on participants’ intentions and efforts to change their prescribing practices. On completing the module the number of participants intending to prescribe fewer BZDs almost doubled. The percentage of participants reporting trying (or to have been trying for some time) to prescribe less sleep medication almost tripled on the longer term.

More relevant however, is the significant and enduring impact on participants’ self-efficacy beliefs. There was an enduring and remarkable decrease in participants reporting not knowing how to meet their goal of prescribing less BZDs. When starting the module 43.1% reported not knowing how to prescribe less sleep medication, compared to only 1.7% on the longer term.

Furthermore, analyses showed a significant effect on the number of participants reporting success in minimizing BZD prescribing. When starting the module, only 12.2% reported success, while several months after completing the E-module, 62.9% reported successfully minimizing BZD prescribing.

Finally, months after the intervention 85.1% of the participants stated that the module changed their prescribing practice and participants reported implementing several demonstrated, non-pharmacological interventions in their practice (Table 3).

In particular, the ‘ICE model of communication’ (82.4%) and the booklet on sleep hygiene education (61.9%) were frequently used and unanimously considered useful by participants. Also the ‘stress-vulnerability model’, ‘the sleep wake diary’ and the booklet on stimulus control therapy were frequently implemented and considered practically useful by most participants. ‘The ABC model’ was the non-drug intervention which was least often implemented, but this was also used by almost half the participants. Most respondents (53%) also considered ‘the ABC model’ practically useful, although opinions were divided.

## Discussion

### Main findings

We were able to document a significant impact of an E-intervention on determinants of BZD prescribing among GPs in vocational training. Furthermore, a lasting impact on participants’ psychological dynamics and their readiness to adhere to prescribing guidelines was documented. This long-term impact is the more remarkable since participants were practicing GPs, encountering many situations in which they were confronted with the tension between wanting to minimize BZD prescribing and feeling a responsibility to help ‘deserving’ patients. Moreover, participants reported experimenting with several demonstrated, non-drug interventions within these complex consultations.

Observed changes in participants’ attitudes and self-efficacy beliefs remained while barriers often cited in the literature [11] remained absent: lacking time for non-drug interventions or perceiving these to be the task of other professionals were not observed during the intervention nor did they arise when participants tried non-drug interventions in the period after the intervention. Anticipating difficulties motivating patients to accept non-drug treatment was a barrier that diminished in the short term, but this effect was not enduring. Although research shows that GPs’ perceptions about patients’ expectations concerning sleep problems tend to be assumed rather than being the product of an actual dialogue [31], the reemergence of this barrier might point out a reality and not merely a perception. We consider this observation an argument for choosing multi-faceted interventions when aiming to improve prescribing and use of BZDs: targeting both prescribers and consumers appears to be the most successful strategy [18].

Moreover, the participation rate in our study demonstrates that many full time working GPs in vocational training are motivated to participate in an e-intervention to learn about BZD prescribing and manage psychosocial consultations. They engaged in several hours of learning, spread over several weeks during the intervention and passed several assessments. This suggests a sense of responsibility for BZD prescribing practice and an appreciation of the e-learning modality among these young GPs.

### Strengths and limitations

Despite guidelines and several campaigns to change prescribing behavior, BZDs continue to be overprescribed and new approaches to improve BZD prescribing practice are needed. The intervention described is innovative in several ways. Firstly, it is a web-based and GP-tailored intervention that is attuned to GPs’ ambivalent attitudes, perceptions, and self-efficacy beliefs. Also, the presented intervention focuses on avoiding initial BZD prescriptions and using psychological treatments as an alternative. It is promising that we were able to document a significant and long-term impact of this e-intervention.

Secondly, it has been agreed that changing the behavior of health professionals is challenging and literature provides little information to guide the choice of interventions aimed at doing so. We demonstrate that a theoretical and psychological understanding of underlying processes helps to guide the development of effective interventions, as Michie et al. [19] argue. Interventions require more than good content: using behavior change techniques and tailoring interventions to user characteristics are vital to promote behavior change, including within internet delivered interventions [32]. Understanding the behavior of healthcare professionals allows interventions to be attuned to their psychological dynamics instead of being limited to simply transferring knowledge. However, interventions aimed at changing health professionals’ behavior tend to be rather preachy. This is ironic since it is well known that preachy approaches are not very helpful for achieving behavioral change in patients.

Thirdly, our study highlights e-learning can be used effectively to attune to GPs and modify their attitudes, perceptions and self-efficacy beliefs.

Fourthly, we demonstrated educational interventions targeting health professionals can be evaluated by selecting relevant psychological determinants as outcome measures. Thinking beyond decreased BZD prescriptions, we were able to document the effectiveness of the intervention through durable changes in several psychological determinants of prescribing practice.

Voluntary participation suggests a sense of responsibility for BZD prescribing practice among our participants. Also the absence of some negative attitudes concerning non-drug interventions was remarkable. Whether these features distinguish included participants from other GPs in vocational training is unknown. Also, reasons for dropping out of our study remain unknown. Thus a selection bias must be taken into account, certainly since participants’ motivation for engaging in this intervention and several assessments, is probably linked to the questioned attitudes and barriers. However, a participation of 33%, puts this in perspective.

The effectiveness of similar interventions with experienced GPs is unknown and there are several arguments to study the effectiveness of this e-intervention with other groups of GPs. Since educational interventions (targeting GPs) that do not rely upon voluntary participation tend to achieve a larger reduction in BZD prescribing [18], this could guide further research. Investigating which mechanisms of change (such as user characteristics) are responsible for the observed effectiveness could help to refine and improve future interventions.

We were not able to register the impact of the e-intervention on rates of BZD prescribing. We were merely able to document GP characteristics that are likely to lead to better prescribing practices. Since people do not always do what they want to do or think they do, there will always remain a gap between psychological determinants, reported behavior and actual behavior. Nevertheless, when aiming to bring about enduring behavior change, it is essential to target mechanisms of change [32]. For example in antibiotic prescribing, the effectiveness of similar theoretical based and GP-tailored interventions on actual prescribing behavior has been documented [33].

## Conclusion

In conclusion, this study documents a significant and long-term impact of a theoretical based e-intervention on determinants of BZD prescribing in GPs in vocational training. Our GP-tailored intervention significantly changed several relevant psychological determinants of BZD prescribing and had an impact on participants’ readiness to adhere to prescribing guidelines. Significant changes in attitudes and self-efficacy beliefs remained in the long-term, while participants encountered many situations in which they were confronted with the complex task of managing psychosocial consultations and in which they experimented with demonstrated non-drug interventions.

## Additional file

Additional file 1: e-intervention: prescribing BZDs. This additional file contains more details about the e-intervention that focuses on avoiding initial BZD prescriptions and using psychological interventions as an alternative. (DOCX 22 kb)

## Abbreviations

BZD: Benzodiazepine
GP: General Practitioner
SDT: Theory of Self-Determination
TTM: Transtheoretical model
