# Supplementary material for: Teaching young GPs to cope with psychosocial consultations without prescribing: a durable impact of an e-module on determinants of benzodiazepines prescribing
Source: BMC Med Educ. 2017 Dec 19;17:259. doi: 10.1186/s12909-017-1100-3 (PMC5735912; doi:10.1186/s12909-017-1100-3)
Supplement: Additional file 1: — e-intervention: prescribing BZDs. This additional file contains more details about the e-intervention that focuses on avoiding initial BZD prescriptions and using psychological interventions as an alternative. (DOCX 22 kb) [file 12909_2017_1100_MOESM1_ESM.docx]

# e-intervention: prescribing BZDs

| Avoiding initial BZD prescription and using alternative, non-pharmacological treatment strategies for patients suffering insomnia was the core focus of the intervention. Specifically, the module aspired to address to GPs ambivalent attitudes and perceptions, which are known to determine their prescribing practices [11] and not merely focus on GPs acquiring knowledge.  The e-module could be accessed online and participants could choose freely whether they completed the module at once or in several stages. Though some suggestions were offered by the authors, participants were also given the autonomy to choose the order and amount of time they spent on several items and chapters. The module was developed within a familiar format for GPs, creating a feeling of relatedness: participants met several virtual patients who were waiting for them in the waiting room of a general medical practice. Through video-consultations, trainees were shown the pitfalls of BZD prescriptions and how non-pharmacological alternatives can be used within the consultation room. The e-module provided training in enhanced communication skills. GPs were also provided with several tools and documents that could support them.   - The ‘ICE model of communication’ encourages GPs to explore patients ideas, concerns, and expectations about diagnosis and/or treatment (the ICE acronym) and exemplifies a patient-centered approach [29]. - ‘Sleep hygiene education’ refers to educating patients about healthy sleep behaviors and sleep-conducive environmental conditions [30,31]. - The ‘Stress-vulnerability model’ (diathesis–stress model) is a model that suggests that people inherit a predisposition to mental illness, but this ‘vulnerability’ in itself is not sufficient to manifest the disorder and requires interaction with bio)psychosocial stressors [32]. - A ‘Sleep wake diary’ is an assessment tool to explore patients’ sleep/wake patterns. This registration allows GP and patient to tailor interventions such as ‘sleep hygiene education’ or ‘stimulus control therapy’. - ‘Stimulus control therapy’ is a classical conditioning approach to re-associating the bed and bedroom with successful sleep attempts [31]. The ‘ABC Model’ can be used to educate patients about the relationship between thoughts, emotions and behavior, which is an important part of therapeutic change. The model can be used as a registration tool, inviting patients to record sequences of events in terms of ‘Activating events’, ‘Beliefs’ and’ Consequences’ [33].   To encourage reflection, video-consultations were shown in several stages and participants were frequently asked how and why they would act themselves in those particular situations. Rather than telling participants what to do, the module gave participants a chance to experience the consequences of their choices as this enhances a feeling of control, competence and autonomy.  Scientific knowledge and guidelines concerning BZD prescribing was integrated and referred to within the module. However this was not the core material of the E-module, but was stored within a virtual ‘library’, available for participants whenever they wanted to consult some literature. |
| --- |
